# Annotations

**Published:** 1889-05-25

**Authors:** 


### Annotations.

**Cabby's Progress.**

Mr. Darwin has shown, in an infinite variety of ways, how interesting the commonest objects may become to the student if investigated in a scientific spirit. Cabmen are probably as important, at least in the imperial economy of Great Britain, as earthworms or even beetles and crabs; and it is worth while considering scientifically a statement which was made at the annual meeting of the Cabdrivers' Benevolent Association last week. Mr. John Aird, M.P., who presided at the meeting, stated that less than twenty years ago, or, to be more precise, between the years 1871 and 1875, the summonses taken out against cabmen at Marlborough-street alone averaged about fifty per week; whereas at the present time, at the same police-court, the summonses number only fifty-one for the whole year. Mr. Aird thence concluded that the conduct of the cabmen had recently been of a decidedly exemplary character. The questions which it is desirable to have answered on hearing such a statement are something like these: What is the reason, or what are the reasons, of this fifty-fold improvement in so short a space of time? Has education had anything to do with it? The new Education Act was passed about twenty years ago, and has been in operation during the period of the cabmen's marked improvement. It would be worth while to find out what proportion of the present cabdrivers have attended elementary schools. Or has total abstinence been at all an important factor in the production of the change? It would not be difficult for enthusiastic temperance advocates to ascertain whether there are many abstainers among cabmen, and whether the proportion is much greater now than it was between 1871 and 1875. It would also be very instructive to know whether or not the average earnings of the cab-driving class are greater than they were twenty years ago, and whether their home surroundings are improved. All these questions are of first-rate importance, not only to the philanthropist and the religious teacher, but also to the practical statesman. That only one cabman a week should be compelled to answer for his conduct in a police-court to-day, where fifty used to appear twenty years ago, is a change of much too great significance to have come about without a reason. We all congratulate ourselves on the fact, and most of us who use cabs constantly can bear testimony of a very similar character. The cabman is undoubtedly a marked instance of progressive development. What is the nature of the cause or causes which have so speedily brought about so admirable a result?

**Frogs or Beefsteaks?**

Some people we know "eat to live," whilst others "live to eat." The well-conditioned Briton would be ashamed to belong to the latter class. It is considered rather meritorious to despise the pleasures of the table. Young men are especially enjoined to eat moderately, and of the plainest food. In the eyes of many persons the youthful Scotch student who lives on half-a-crown a week, and takes a first-class in theology or medicine, is of all youths the very pink and paragon. There is a little grain of sense and virtue in all that, and only a little grain. The animating motive that prompts it is good, but the directing sense that guides it is almost wholly unscientific and foolish. It is a very elementary principle of logic that tells us there can be nothing in the conclusion which was not previously in the premisses. The cleverest conjuror in the world cannot bring the tiniest hazel nut or even pin out of his miraculous hat unless it has been previously put in, either with exaggerated ostentation or by some secret and cunning legerdemain. So much, at least, is plain and indisputable. But food is the raw material out of which the wonderful machinery of digestion has to produce all manner of products of the most astonishing kind. No food means no raw material; insufficient food means insufficient raw material; coarse and unpalatable food means coarse and unworkable raw material. What is the inevitable result? It is twofold at least, but it may be manifold. Coarse and unworkable raw material must necessarily give a coarse finished product, coarse thought, coarse feeling, coarse looks; and, moreover, it cannot but injure the machinery of digestion. We are all for giving everybody his due, even the Frenchman and the Belgian. Carrying out this principle, it cannot be disputed that in the art of feeding the human animal, "they do these things better in France." The middle-class Englishman eats hunks of beef and wedges of mutton, followed by pudding as solid as lead, and washed down with strong Burton ale, or still stronger Dublin stout. That is very good fare for ploughmen or foxhunters. To the town man it is simply ruin. The Frenchman knows better. Neither in town nor in country will he feed like a *carnivore*. He believes in variety; he likes a little bit of many dishes, not a vast mountain of one. He likes it made pleasant to the sight, and deliciously delicate to the taste. He takes time over it. It is quite the rule on the Continent to see all the busy men in the town spend at least an hour at the restaurant over the mid-day meal. The eating done, a smoke, a game of draughts or dominoes, and a cup of coffee are invoked as aids to digestion. The natural result is that a Frenchman or a Belgian looks happy at meals, and still happier at his coffee and dominoes. Is any man unscientific enough to say that the Frenchman is wrong and the Englishman right? Eating is undoubtedly one of the chief functions of life, and if it be right to make all life bright and happy, then eating should be one of its chief pleasures. We English have a great deal to learn from many countries. We cannot do better than begin by learning how to prepare food so that it shall be pleasant and tempting to the senses, and how to eat it without looking like mutes at a funeral, or lions at an ill-managed "Zoo."

**A Plea for Football.**

"A Young Schoolmaster," whose letter appears in another column, is a little late in the day in entering his "football" protest, inasmuch as the season is practically over and the time of cricket is already upon us. We publish his letter, however, because, although the game itself be confined to the winter months, the public, and especially the school interest in it is perennial. It must not be thought that because football is a school game, therefore it is a school game only. Newspapers, and particularly those so cosmopolitan in their scope as the *Hospital*, owe a duty to the whole population; and if dangerous football accidents do occur, and that in considerable numbers, it is clearly imperative upon us to call attention to the facts, and if changes be demanded, to insist that they be made. It is not denied that a large number of casualties, most of them serious and some of them fatal, occurred during the season just past. Our contention is, and we have been practically familiar with the game for many years, that there is no necessity for such casualties, and that if the present rules, either of school or outside players, permit of the methods of play which result in them, those rules ought undoubtedly to be altered. Is it, however, in accordance with universal experience that dangerous casualties seldom or never take place at school matches or in high-class clubs? By no means! It is, perhaps, true that the two clubs mentioned—viz., the Preston North End and Blackheath—have had no casualties during the past seven or eight months. But are not high-class clubs numbered by the hundred, and ordinary clubs by tens of thousands? What would the history of all the matches of all the clubs of every class reveal, even if taken during only a single season? We do not like to hazard a guess; but if all the minor, all the serious, and all the fatal accidents could be stated in order, it is certain that every parent of schoolboys in Great Britain would be seriously alarmed. Our correspondent suggests that none but certificated players should take part in matches. But that is practically the case already, inasmuch as for every match of any importance the players are carefully selected. And what is the real difference between "selection" and "certification"? The suggestion that heavy fines be inflicted on clubs in which accidents occur is not practicable. So far as we can see the only thing to be done is to alter the rules. Football in itself is a splendid game, and admirably adapted to the genius of young England. There is no fear that it will be abolished, and no reason why it should be. But it can be played in such a way as to call forth all the energy, all the enthusiasm, all the pluck, and all the invincible determination we see at present without exposing young and ardent spirits to imminent danger, and the risk of death. There is plenty of time before the commencement of next season for a judicious reconsideration of the whole question of rules. We repeat the expression of our hope that reason may prevail, and that the game shall be made really a game, and not a life and death struggle.

